# Gender Differences in the Appetite Response to a Satiating Diet

**DOI:** 10.1155/2015/140139

**Published:** 2015-09-09

**Authors:** Alexandra Bédard, Anne-Marie Hudon, Vicky Drapeau, Louise Corneau, Sylvie Dodin, Simone Lemieux

**Affiliations:** ^1^Institut sur la Nutrition et les Aliments Fonctionnels (INAF), Pavillon des Services, Université Laval, 2440 Boulevard Hochelaga, Québec, QC, Canada G1V 0A6; ^2^École de Nutrition, Pavillon Paul-Comtois, Université Laval, 2425 rue de l'Agriculture, Québec, QC, Canada G1V 0A6; ^3^Département de l'Éducation Physique, Pavillon de l'Éducation Physique et des Sports, Université Laval, 2300 rue de la Terrasse, Québec, QC, Canada G1V 0A6; ^4^Département d'Obstétrique et Gynécologie, Pavillon Ferdinand-Vandry, Université Laval, 1050 Avenue de la Médecine, Québec, QC, Canada G1V 0A6

## Abstract

We examined gender differences in appetite sensations when exposed to Mediterranean diet (MedDiet) meals and determined whether there are gender differences in the change in the satiating properties of the MedDiet over time. Thirty-eight men and 32 premenopausal women consumed a 4-week isoenergetic MedDiet under controlled conditions. Visual analogue scales were used to measure perceived appetite sensations before and immediately after each meal consumed over the course of one day (Wednesday) of the first and the fourth week of intervention. Women reported greater decreases for desire to eat, hunger, and appetite score than men in response to the consumption of the MedDiet meals (gender-by-meal interactions, resp., *P* = 0.04, *P* = 0.048, and *P* = 0.03). Fullness and prospective food consumption responses did not significantly differ between men and women. Between the first and the fourth week of intervention, premeal prospective food consumption increased with time in men (*P* = 0.0007) but not in women (*P* = 0.84; *P* for gender-by-time interaction = 0.04). These results indicate gender differences in appetite sensations when exposed to the MedDiet. These results may be useful in order to have a better understanding of gender issues for body weight management.

## 1. Introduction

Taking into consideration the alarming rise in obesity rate observed around the world in last decades [1], the development of efficient strategies for body weight management is now established as a major public health issue. From a nutritional point of view, it is widely recognized that adopting a satiating diet with a low energy density may prevent energy overconsumption through an adequate appetite control [2, 3]. Accordingly the traditional Mediterranean diet (MedDiet) has been recently the subject of increasing attention for its usefulness in body weight management, mainly due to its high fiber content and low energy density, favouring appetite control and, consequently, body weight loss and/or maintenance [4, 5].

The few clinical trials that have documented differences between men and women in appetite response to nutrient manipulations reported gender differences. In fact, results from previous studies suggest that women are more sensitive to overfeeding [6] and macronutrient changes [7], leading to greater changes in appetite sensation ratings and/or subsequent energy intake in women than in men. However, it is difficult to generalize these results since almost all previous studies examined the acute effect of a single nutrient/food on appetite sensations. In fact a given nutrient or food may act differently on appetite sensations depending on interaction with other nutrients and foods present in the diet. In the case of the MedDiet, a meta-analysis of randomized controlled trials has concluded that the adoption of the MedDiet may be a useful tool to reduce body weight [4]. Moreover, a 3-fold greater decrease in BMI was noted in studies conducted with female cohorts (>50% of women) when compared to male cohorts. Considering gender differences in appetite regulation, one possible explanation is that these gender differences are partly due to different appetite responses in men and in women when exposed to a MedDiet. However, no study has up to now investigated this issue. The first objective of the present study was, therefore, to document gender differences in appetite responses when consuming MedDiet meals. In addition, previous studies have documented a habituation phenomenon to food cues, which corresponds to changes in physiological and behavioral responses to foods after repeated consumptions [8]. Thus the second objective was to gain insight into another unexplored issue, namely, to determine whether the satiating properties of the MedDiet persist over time, still with a particular attention for gender differences.

## 2. Participants and Methods

### 2.1. Participants

Participants were men and premenopausal women, from the Québec city metropolitan area (Canada), aged from 24 to 53 years, with a BMI ranging from 22 to 48 kg/m^2^ and characterized by a stable body weight (±2.5 kg) during at least the three months before the study. Participants were recruited through the media and electronic newsletters. Eligibility was determined on the basis of a slightly deteriorated lipid profile, as previously reported [9]. The study protocol was approved by the Laval University Ethics Committee. The present study was conducted according to the guidelines laid down in the 1964 Declaration of Helsinki and its later amendments. All participants provided written informed consent before enrolling in the study. This clinical trial was registered at https://www.clinicaltrials.gov/ as NCT01293344.

One hundred and forty-four individuals volunteered to the study and 75 subjects met the inclusion criteria. Among this initial group, five subjects dropped out during the run-in period for personal reasons (four men and one woman). Therefore, 38 men and 32 women consumed the 4-week MedDiet.

### 2.2. Study Design

A 4-week run-in period preceded the feeding intervention in order to minimize the intra- and intervariability in dietary intakes. During this run-in period, participants received personalized recommendations by a registered dietitian in order to follow the recommendations of the Canada's Food Guide [10]. As previously shown, this run-in period led to similar dietary habits between men and women in the month prior the feeding intervention [9].

The fully controlled feeding intervention was undertaken as a parallel design in which both men and women were assigned to a 4-week experimental MedDiet. All foods and drinks were prepared by food technicians at the Clinical Investigation Unit at the Institute of Nutrition and Functional Foods (Laval University) and were provided to participants. A 7-day cyclic menu was used and repeated for 4 weeks (see Table A.1 in Supplementary Material available online at http://dx.doi.org/10.1155/2015/140139). That cyclic menu allowed us to provide the same menu for each particular day of the week (e.g., the menu was exactly the same on each Wednesday during the feeding intervention).  Table 1 presents the numbers of servings of key foods consumed daily during the experimental MedDiet, as previously reported [9, 11, 12].  Table 2 shows the nutritional composition of the experimental MedDiet, as previously reported [9, 11, 12]. Before the consumption of the MedDiet, the habitual energy intake of each participant was established by averaging the energy requirements estimated by a validated food frequency questionnaire (FFQ) [13] and energy needs as determined by the Harris-Benedict formula. On each weekday, body weight was measured immediately before lunch and foods and energy provided were revised if necessary for minimizing body weight fluctuations.

### 2.3. Subjective Appetite Sensations

Visual analogue scales (VAS) were used to measure subjective appetite sensations before and immediately after each meal consumed on the Wednesday of the first (*T* = 1) and fourth (*T* = 4) feeding weeks. In order to respect their usual prandial schedule, participants were free to choose the moment at which they consumed their meals throughout the day. No snack was provided to participants. VAS (0–150 mm) were composed of four questions adapted from Hill and Blundell [14]: how strong is your desire to eat? (very weak–very strong), how hungry do you feel? (not hungry at all-as hungry as I have ever felt), how full do you feel? (not full at all-very full), and how much food do you think you could eat? (nothing at all-a large amount). These VAS measured perceived appetite sensations for, respectively, “desire to eat,” “hunger,” “fullness,” and “prospective food consumption” at this specific moment. Another VAS filled only after meals assessed the liking of meals: did you like the meal? (not at all-very much). Subjects were asked to position a vertical bar on the scale to indicate how they felt with regard to each specific question.

An average score of the four appetite sensations was also calculated using the following formulae: (desire to eat + hunger + (150 − fullness) + prospective food consumption)/4. This appetite score indicates the overall perceived appetite sensation, as used in other studies [15, 16].

The satiating capacity of the meal was also assessed by the satiety quotient, a concept adapted from Green and collaborators [17] and thereafter used by other research teams [18–21]. The satiety quotient (mm/100 kcal) was determined for each meal using the following equation: ((postmeal fullness − premeal fullness)/(energy content of the test meal)) *∗* 100. A lower satiety quotient represents a weaker satiating capacity of the meal.

### 2.4. Eating Behavioral Traits

A score was determined for dietary restraint (conscious control of food intake with concerns about shape and weight), disinhibition (overconsumption of food in response to a variety of stimuli associated with a loss of control on food intake), and susceptibility to hunger (food intake in response to feelings and perceptions of hunger) at the beginning of the feeding intervention using the 51-item validated Three-Factor Eating Questionnaire [22, 23]. These three eating behavioral traits were further divided into more specific subscales [24, 25] as previously described [26].

### 2.5. Physical Activity Participation

Daily energy expenditure from physical activity participation was determined using a validated 3-day activity diary record developed by Bouchard et al. [27], as previously reported [26]. Subjects were instructed to maintain stable physical activity participation during the study protocol.

### 2.6. Anthropometric Measurements

Body weight, height, and body mass index (BMI) were measured using standardized methods [28].

### 2.7. Statistical Analyses

A *P* ≤ 0.05 (two-sided) was considered significant. Gender differences in characteristics at the beginning of the controlled feeding intervention (i.e., after the run-in period) were assessed by the Student's *t*-test for unpaired data (SAS version 9.2, SAS Institute Inc., Cary, NC, USA).

In order to reach the first objective (i.e., to document gender differences in appetite sensation responses to the consumption of Mediterranean meals), we measured appetite sensations during the fourth week of the feeding MedDiet intervention (*T* = 4). At this time, one man and one woman did not fill out their VAS; therefore, 37 men and 31 women were included in the analyses. MIXED procedures for repeated measurements were used to assess main effects of gender (men versus women), meal (i.e., before versus after meal), meal type (i.e., breakfast, lunch, and dinner), and their interactions on appetite sensations as well as main effects of gender, meal type, and gender-by-meal type interaction on the satiety quotient. When a significant main effect was detected, pairwise differences between and within group means were tested with the Tukey-Kramer adjustment. Associations between mean satiety quotient (i.e., the mean value for breakfast, lunch, and dinner) and energy intake were assessed by Pearson's correlation analyses. The statistical software MedCalc version 12.4.0 (Acacialaan 22, B-8400 Ostend, Belgium) was used to analyze gender differences in the correlation coefficients.

For the second objective (i.e., to determine whether the satiating properties of the MedDiet persist over time in men and women), we measured appetite sensations during both the first (*T* = 1) and the fourth (*T* = 4) feeding weeks. In the MIXED procedures, gender (men versus women), time (i.e., first week versus fourth week), meal type (i.e., breakfast, lunch, and dinner) and their interactions were entered as main effects. Gender-by-time interactions were used to investigate whether changes in appetite sensations over time were different between men and women. Statistical analysis including appetite sensations at both *T* = 1 and *T* = 4 was adjusted for change in energy intake between the two measurements (men +216 ± 301 kcal and women +105 ± 180 kcal, *P* for gender difference = 0.06).

For both objectives, similar results were obtained for breakfast, lunch, and dinner. Therefore, all data are provided as estimated means of all meals combined from the linear mixed-effects model with their standard errors.

## 3. Results

### 3.1. Participant Characteristics

As previously reported [9], there was no significant difference between men and women for mean age and BMI (Table 3). As expected, men had higher body weight and daily energy needs than women. Energy expenditure related to physical activity was also higher in men than in women. Eating behaviors were similar between genders, except for emotional susceptibility to disinhibition, for which higher values were observed in women.

### 3.2. Appetite Sensation Responses to MedDiet Meals

**Figure 1:**
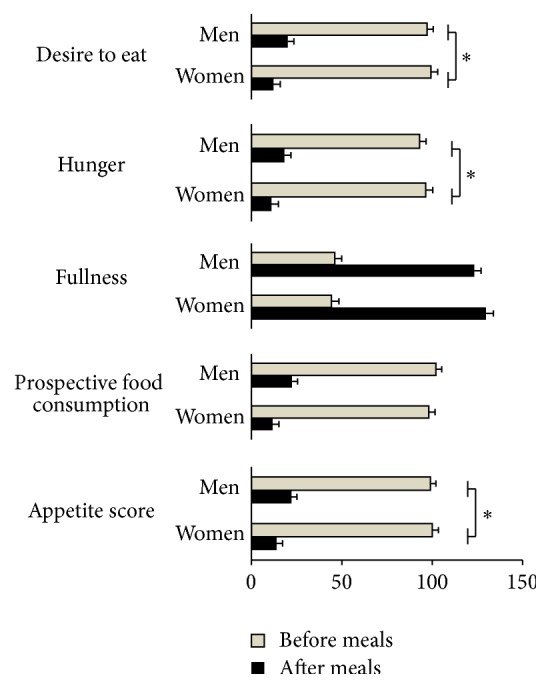
Appetite sensations before and after consumption of the Mediterranean meals during the fourth week of the feeding intervention. All data are provided as estimated means of all meals combined from the linear mixed-effects model with their standard errors. ^*∗*^ Women reported more decreases for their desire to eat, hunger, and appetite score in response to the consumption of the Mediterranean meals than men, *P* < 0.05.

Ratings of appetite sensations before and after meals, measured during the fourth week of intervention, are presented in Figure 1. First, there was no gender difference in premeal appetite sensations (between-gender differences: desire to eat, *P* = 0.98; hunger, *P* = 0.92; fullness, *P* = 0.99; prospective food consumption, *P* = 0.84; appetite score, *P* = 1.00). As expected, decreases in desire to eat, hunger, prospective food consumption, and appetite score and increases in fullness sensations were observed in response to MedDiet meals in both men and women (*P* < 0.0001). However, women reported greater decreases in desire to eat, hunger, and appetite score than men in response to the consumption of the MedDiet meals (−87.7% for women and −79.3% for men for the desire to eat, *P* for gender-by-meal interaction = 0.04; −88.5% for women and −80.3% for men for the hunger, *P* for gender-by-meal interaction = 0.048; and −86.1% for women and −77.6% for men for the appetite score, *P* for gender-by-meal interaction = 0.03). Fullness and prospective food consumption responses did not significantly differ between men and women (gender-by-meal interaction effects, resp., *P* = 0.15 and *P* = 0.12).

**Figure 2:**
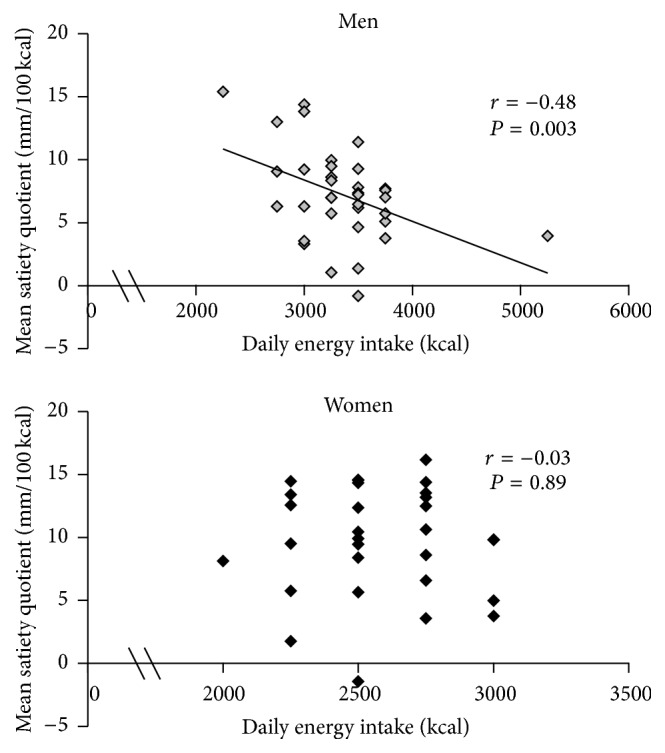
Pearson's correlations between the mean satiety quotient for fullness sensation (i.e., the mean value for breakfast, lunch, and dinner) and daily energy intake in men and women during the Mediterranean diet feeding intervention.

Gender differences were found for satiety quotients, women having higher values than men (men: 7.1 ± 0.6 mm/100 kcal; women: 9.7 ± 0.8 mm/100 kcal; *P* for gender effect = 0.006). However, these gender differences were no longer significant after adjustment for daily energy intake (*P* for gender effect = 0.80). Mean satiety quotient was associated with energy intake in men, but not in women (gender differences for the correlation coefficients, *P* = 0.05) (Figure 2). A similar association was obtained in men after the exclusion of two outliers for energy needs (daily energy intake of 2250 kcal and 5250 kcal, *r* = −0.35 and *P* = 0.04).

### 3.3. Changes in Appetite Sensations over Time

For premeal appetite sensations, a gender difference was found for prospective food consumption (*P* for gender-by-time interaction = 0.04), and only men reported increases over time (+16.5%, *P* = 0.0007 for men and +3.5%, *P* = 0.84 for women; Table 4). Increases in desire to eat and appetite score were reported over time (mean change in men and women combined: +8.1% for desire to eat and +7.1% for appetite score; *P* for time effect = 0.01 and 0.009). No gender difference was found for these two appetite sensations (*P* for gender-by-time interactions = 0.41 and 0.36). No change was observed for premeal hunger and fullness over time in both men and women.

For postmeal appetite sensations, no gender difference was reported for any of the appetite sensations measured, as suggested by nonsignificant gender-by-time interactions (Table 4). However, increases in desire to eat and hunger were reported (mean change in men and women combined: +11.2% for desire to eat and +26.3% for hunger; *P* for time effect = 0.03 and 0.01). No change was observed for postmeal fullness, prospective food consumption, and appetite score over time in both men and women.

Satiety quotients were similar during the first and the fourth weeks for both men and women (*P* for time effect = 0.56; *P* for gender-by-time interaction effects = 0.10; Table 4).

### 3.4. Subgroup Analyses

Both men and women had a small body weight loss (1.3 kg or 1.4% of initial body weight in men and 0.5 kg or 0.7% in women). This change in body weight occurred mainly in the first week of the feeding intervention (body weight loss of 0.80 kg in men and 0.45 kg in women). Adjustment for body weight changes did not influence any of the results obtained.

Moreover, in order to ensure that change in body weight did not influence results obtained, we repeated analyses within a subgroup of men (*n* = 13) and women (*n* = 19) who maintained their body weight stable during the MedDiet intervention (<1% of body weight change, body weight loss of 0.17 ± 0.57% in men and 0.17 ± 0.55% in women). These analyses revealed that the magnitude of differences between men and women in appetite sensation responses to MedDiet meals and in changes in appetite sensations over time with the MedDiet remained unchanged as compared with those noted when the whole sample was included in the analyses (not shown).

### 3.5. Liking Ratings for MedDiet Meals

Similar liking ratings for MedDiet meals were noted in men and women at the fourth week (gender difference, *P* = 0.22). The liking ratings did not change over time (*P* for time effect = 0.59) in both men and women (*P* for gender-by-time interaction = 0.77; first week: 114.5 ± 3.5 mm for men and 125.3 ± 3.8 mm for women; fourth week: 116.4 ± 3.4 mm for men and 125.9 ± 3.8 mm for women).

## 4. Discussion

In a controlled eating context in which men and women consumed exactly the same diet, our results showed that, although they had similar appetite sensations before meals, women reported larger reductions in desire to eat, hunger, and overall appetite score than men when exposed to MedDiet meals. Moreover, when we analyzed gender differences in changes in appetite sensations over time, results showed that only men increased their premeal prospective food consumption between the first and the fourth weeks of the feeding intervention. However, the satiating capacity of each calorie of the MedDiet meals was stable across time in both men and women, as suggested by similar satiety quotients in the first and the fourth week of intervention in both sexes. Taken together, these results indicate gender differences in appetite responses when consuming MedDiet meals.

Women's appetite sensations decreased more in response to the consumption of the Mediterranean meals than men. Westerterp-Plantenga et al. [7] observed greater changes in perceived satiety sensations in women than in men in response to a diet relatively high in protein (30% of energy) compared to a lower protein diet (10% of energy) when energy intake was held constant. Moreover, our results are in concordance with some studies which reported higher postmeal satiety ratings in women than in men [29–31]. Gender differences in many appetite-related areas have been previously highlighted, which may give insight about reasons for the higher satiating effect in women than in men. According to these previous studies, these gender differences may be partly due to physiological regulation of appetite through sex hormones. More specifically, female sex hormones, mainly estrogens, influence central and peripheral signals from some hormones implicated in feedback controls of eating, including ghrelin, cholecystokinin, insulin, and leptin and may mediate the estrogenic inhibition of eating during the consumption of a meal as highlighted in previous human studies [32]. In addition, Cornier et al. [29] observed that women have increased neuronal activation in response to food cues and have a greater attention, cognitive processing, and inhibitory response to food cues than men. These differences in brain activation in response to food stimuli may then consequently produce gender specific appetite responses. Finally, it is also possible that external influences may differentially affect appetite in men and in women (e.g., social and cultural roles, emotional issues, and dietary behaviors). Since our study was not elaborated to document mechanisms behind these gender differences, additional studies are needed in order to shed light on reasons for these gender differences in the appetite response when exposed to MedDiet meals.

In a meta-analysis of randomized controlled trials aiming at increasing the adherence to the MedDiet, Esposito and collaborators have observed a 3-fold greater decrease in BMI in studies conducted with female cohorts compared to male cohorts [4]. Results from the present study suggest that the greater decrease in BMI in response to the MedDiet interventions may be partly due to a greater satiating effect in women than in men. However, because of our study design in which participants were held at a constant body weight, it is not possible to determine whether gender differences in perceived appetite sensation responses observed in the present study would translate into significant differences in spontaneous food intake. Therefore, although a satiety-based approach has been suggested for body weight management [33] and that reported appetite sensations have been identified as good predictors of subsequent energy intake in men and women [18], the present results should not be overinterpreted and further studies need to determine whether the adoption of a satiating diet such as the MedDiet is a more useful strategy for body weight management in women than in men.

Gender differences were noted for the satiety quotient, suggesting that one calorie from the MedDiet is more satiating for a woman than for a man. This result was expected since energy needs are habitually higher in men than women (3382 ± 489 kcal in men and 2570 ± 264 kcal in women in this study). Gender differences in the satiety quotient disappeared after adjustment for energy intake. These results highlight that differences in energy needs have to be taken into account when assessing gender differences in the satiating effect of meals/diet with the satiety quotient. Similarly, this issue may also be considered in other contexts in which energy need disparities exist (e.g., context in which subjects decrease their energy needs with body weight loss).

A negative association between the satiety quotient and energy needs is expected; that is, the more energy needed, the lesser the satiating effect of each calorie. This negative association was found in men, but not in women. The lack of a clear association in women might be due to the fact that appetite response to an eating episode is a complex process including both internal and external signals. Previous studies have suggested that men eat more according to their internal, biological hunger signals than women, who are more influenced by external, situational, and emotional signals [34].

As previously stated, a habituation phenomenon to food cues may occur after repeated consumptions versus a new food [8]. Therefore, it was relevant to document whether the satiating properties of the MedDiet persist in the medium term in non-Mediterranean men and women. Our results demonstrated that the satiating effects of the MedDiet within a meal persist over time in either sex. In fact, as suggested by the satiety quotient, the satiating capacity of each calorie in the first week was similar to the one reported in the fourth week. However, increases in desire to eat and overall appetite score before meals and in desire to eat and hunger after meals were reported in both men and women. Moreover, an increase in premeal prospective food consumption has been noted, but only in men. Increased appetite has been suggested as a barrier to body weight management [33, 35]. Moreover, although both perceived appetite sensations and satiety quotient have been associated with the subsequent energy intake [18, 36, 37], perceived appetite sensations measured before and after meals were found to be the strongest predictors of* ad libitum* test lunch energy intake [18]. Overall these results suggest that energy intake could be increased over time with the MedDiet. However, the MedDiet is a food pattern with a low energy density and high satiating properties; therefore, a large amount of food had to be consumed by subjects of the present study in order to maintain constant their body weight. Consequently, another possibility is that participants felt uncomfortable at the beginning of the study given the large amount of food they had to consume and that appetite sensations reported at the fourth week are more similar to those experienced in a real-life context and more in line with the usual comfort sought. Since the gastrointestinal comfort of participants has not been evaluated in the present study, it is not possible to draw conclusion about this aspect of appetite regulation.

Although the intervention aimed at being isoenergetic, both men and women experienced a small body weight loss. As several studies have demonstrated that changes in body weight may influence appetite sensations [18, 38], it was, therefore, important to ensure that results obtained were not influenced by this small body weight loss. Accordingly additional analyses showed that adjustment for body weight changes did not influence any of the results obtained. Therefore, these results suggest that the observed impact of the MedDiet meals on appetite sensations in men and women was independent of this slight body weight loss.

We are aware that the absence of a comparison diet may be viewed as a limitation, which limits the conclusions on true treatment effects. However, the satiating proprieties of the MedDiet and its food components have been widely documented in the past years [4, 5]. Therefore, this study did not have as an objective to demonstrate the satiating effects of the MedDiet but rather to investigate gender differences in appetite responses obtained during the consumption of the MedDiet, which was possible since men and women consumed exactly the same diet.

## 5. Conclusions

In summary, our study highlighted the existence of gender differences in appetite sensation responses when consuming MedDiet meals. Moreover, even if both men and women desire to eat, hunger, and appetite score over time on the MedDiet increased, only men reported an increase in prospective food consumption before meals. These results highlight the importance of systematically considering gender issues when assessing the effects of the diet on appetite control. As a next step, further studies need to investigate whether these gender differences in perceived appetite sensations are reflected by differences in* ad libitum* food intake and body weight management.

## Supplementary Material

Table A.1 shows the 7-day cyclic menu used during the controlled Mediterranean diet intervention. All foods and drinks were prepared by food technicians at the Clinical Investigation Unit at the Institute of Nutrition and Functional Foods (Laval University) and were provided to men and women. That cyclic menu allowed us to provide the same menu for each particular day of the week (e.g. the menu was exactly the same on each Wednesday during the feeding intervention).

## Figures and Tables

**Table 1:** Servings of key foods of the Mediterranean pyramid consumed daily during the experimental Mediterranean diet phase for a 10 460 kJ/d (2500 kcal/d) menu.

Key foods^a^	MedDiet (servings/d)
Olive oil (mL)	43.3
Whole grains products	5.7
Fruits and vegetables	16.1
Legumes	0.5
Nuts	0.9
Cheese and yogurt	2.0 Mostly low in fat
Fish	1.3
Poultry	0.9
Eggs	0.3
Sweets	0.3
Red meat	0.2
Red wine	1.3

MedDiet: Mediterranean diet.

^a^Extra virgin and virgin olive oils were used. Serving size for whole grains products = 125 mL (rice, pasta, bulgur, and couscous), one bread piece or 30 g cereal; serving size for fruits and vegetables = 125 mL; serving size for legumes = 175 mL and for nuts = 30 g; serving size for fish, poultry, and red meat = 75 g; serving size for egg = 100 g; serving size for dairy products (mostly low fat cheese and yogurt) = 50 g cheese, 175 g yogurt, and 250 mL milk; serving size for red wine = 150 mL.

This table has been previously published in other publications [9, 11, 12].

**Table 2:** Daily nutritional composition of the experimental Mediterranean diet for a 10 460 kJ/d (2500 kcal/d) menu.

	MedDiet for 10 460 kJ/d (2500 kcal/d)
Energy (kJ)	10 460
Carbohydrate (% of total energy)	46.0
Fiber (g)	42.3
Protein (% of total energy)	17.0
Fat (% of total energy)	32.0
SFA (% of total energy)	6.7
MUFA (% of total energy)	18.1
PUFA (% of total energy)	4.7
Cholesterol (mg)	289.7
Alcohol (% of total energy)	5.0
MUFA to SFA ratio	2.7
Sodium (mg)	3039

MedDiet: Mediterranean diet.

This table has been previously published in other publications [9, 11, 12].

**Table 3:** Characteristics of men and premenopausal women before the 4-week fully controlled Mediterranean diet intervention.

	Men (*n* = 38)		Women (*n* = 32)		Gender difference
	Mean	SEM	Mean	SEM	*P* value^a^
Age (years)^b^	42.6	1.2	41.2	1.3	0.42
Body weight (kg)^b,c^	91.6	2.2	78.0	2.6	**<0.0001**
BMI (kg/m^2^)^b,c^	29.0	0.5	29.6	1.0	0.87
Daily energy needs (kcal)^c^	3169	493	2476	261	**<0.0001**
Energy expenditure from physical activity (kcal/kg·day)^c,d^	4.96	1.01	1.88	0.53	**0.009**
Restraint	7.7	0.6	8.4	0.7	0.46
Rigid restraint	2.2	0.3	2.3	0.3	0.69
Flexible restraint^e^	2.3	0.2	2.9	0.3	0.06
Disinhibition	6.0	0.4	6.8	0.5	0.18
Situational susceptibility to disinhibition	2.8	0.2	2.9	0.3	0.80
Emotional susceptibility to disinhibition	0.8	0.2	1.5	0.2	**0.008**
Habitual susceptibility to disinhibition^c,f^	0.7	0.2	0.8	0.2	0.60
Susceptibility to hunger	4.7	0.6	4.3	0.5	0.58
Internal hunger^c^	1.9	0.3	1.3	0.3	0.15
External hunger	1.9	0.3	2.0	0.2	0.88

Dietary restraint, score 0 to 21; disinhibition, score 0 to 16; susceptibility to hunger, score 0 to 14; rigid and flexible restraint, score 0 to 7; situational and habitual susceptibility to disinhibition, score 0 to 5; emotional susceptibility to disinhibition, score 0 to 3; and internal and external hunger, score 0 to 6. A higher score represents a higher level of this particular eating behavioral trait.

^a^Differences between men and premenopausal women were assessed by Student's *t*-test.

^b^These characteristics have been reported in a previous publication [9].

^c^Analysis was performed on transformed values.

^d^Physical activity level: missing value for one man and one woman.

^e^Flexible restraint: missing value for one man.

^f^Habitual susceptibility to disinhibition: missing value for one man.

**Table 4:** Appetite sensations at the first (*T* = 1) and fourth (*T* = 4) feeding weeks in men and women^a^.

	Men (*n* = 38)				Women (*n* = 32)				Time^b^	Gender-by-time^b^
	*T* = 1		*T* = 4		*T* = 1		*T* = 4			
	Mean	SEM	Mean	SEM	Mean	SEM	Mean	SEM	*P* value	*P* value
Mean appetite ratings before meals (mm)										
Desire to eat	87.7	5.1	97.3	5.1	94.0	5.6	99.0	5.6	**0.01**	0.41
Hunger	87.4	5.1	92.7	5.2	91.4	5.7	96.5	5.6	0.08	0.97
Fullness	51.6	5.0	45.5	5.0	46.1	5.6	43.8	5.5	0.17	0.53
Prospective food consumption	87.3	3.9	101.6^**∗**^	3.9	95.0	4.4	98.4	4.3	**0.002**	**0.04**
Appetite score	90.2	4.3	99.1	4.3	96.0	4.8	100.4	4.7	**0.009**	0.36
Mean appetite ratings after meals (mm)										
Desire to eat^c^	14.1	2.4	20.2	2.4	14.9	2.8	12.1	2.7	**0.03**	0.10
Hunger^c^	11.5	2.3	18.2	2.3	11.8	2.6	11.2	2.6	**0.01**	0.12
Fullness	124.6	3.4	122.8	3.4	131.1	3.8	129.8	3.7	0.48	0.91
Prospective food consumption^c^	16.5	2.4	22.3	2.4	10.0	2.7	11.2	2.6	0.13	0.46
Appetite score^c^	16.9	2.3	22.1	2.3	13.9	2.6	13.7	2.5	0.12	0.41
Mean satiety quotient (mm/100 kcal)	7.0	0.6	7.1	0.6	10.5	0.7	9.8	0.7	0.56	0.10

^*∗*^Increase in prospective food consumption was observed only in men, *P* = 0.0007.

^a^There was no meal type by time effect for any of the appetite sensations for which a time or gender-by-time interaction was found. Consequently, all data are provided as estimated means of all meals combined from the linear mixed-effects model with their standard errors.

^b^Statistical analyses were performed with MIXED procedures.

^c^Statistical analysis was performed on transformed values.
