# Supplementary material for: Gender Differences in the Appetite Response to a Satiating Diet
Source: J Obes. 2015 Sep 9;2015:140139. doi: 10.1155/2015/140139 (PMC4579320; doi:10.1155/2015/140139)
Supplement: Supplementary file 1 — Table A.1 shows the 7-day cyclic menu used during the controlled Mediterranean diet intervention. All foods and drinks were prepared by food technicians at the Clinical Investigation Unit at the Institute of Nutrition and Functional Foods (Laval University) and were provided to men and women. That cyclic menu allowed us to provide the same menu for each particular day of the week (e.g. the menu was exactly the same on each Wednesday during the feeding intervention). [file 140139.f1.docx]

| **Meals** | **Monday** | **Tuesday** | **Wednesday** | **Thursday** | **Friday** | **Saturday** | **Sunday** |
| --- | --- | --- | --- | --- | --- | --- | --- |
| **Breakfast** | MÜSLIX Cereal | Whole grain bread | Bran muffin | MÜSLIX Cereal | Bran muffin with raisins | Whole grain bread | Whole grain bread |
|  | Plain Yogurt | Cottage cheese | Plain Yogurt | Plain Yogurt | Plain Yogurt | Cottage cheese | Omelet |
|  | Cantaloupe | Oranges | Strawberries and mango | Dates | Rasberries and blueberries | Honeydew melon | Grapefruit |
|  | Almonds | Almonds | Pistachios | Almonds |  | Marmalade and margarine | Jalsberg cheese |
|  | | | | | | | |
| **Lunch** | Shrimp rice | Moussaka | Lemon chicken | Cod with grilled tomatoes | Frittata | White Kidney bean soup | Mushroom and Almond chicken |
|  | Greek Salad | Green salad and vinaigrette | Leek soup | Asparagus and orange salad | Couscous salad | Quinoa salad | Artichoke salad |
|  |  | Wheat Baguette | Peppers, zucchini and brown rice | Vegetable couscous | Vegetable soup and bread | Whole grain bread | Green beans and brown rice |
|  | Red grapes | Dried Fruits | Fruit Salad | Almond cake | Green grapes | Fruit Salad | Honeydew melon |
|  | | | | | | | |
| **Dinner** | Chicken cacciatore and broccoli | Trout and green beans | Greek Tilapia grilled peppers | Pasta Primavera | Tuna pasta | Chicken with vegetables | Chick pea and vegetable couscous |
|  | Butternut squash soup | Vegetable salad | Spinach salad | White Kidney bean salad | Broccoli and cauliflower salad | Cheese and nut salad | Tomato and pepper soup |
|  | Potatoes | Mushroom Risotto | Bulgur |  |  |  |  |
|  | Fruit Salad | Honeydew melon | Green grapes | Oranges | Dates | Cantaloupe | Baklava |
| **Red wine with each dinner of the week** | | | | | | | |

**Table A.1:** The 7-day cyclic menu used during the controlled Mediterranean diet intervention
